# Reversal of Cocaine-Conditioned Place Preference through Methyl Supplementation in Mice: Altering Global DNA Methylation in the Prefrontal Cortex

**DOI:** 10.1371/journal.pone.0033435

**Published:** 2012-03-16

**Authors:** Weiping Tian, Mei Zhao, Min Li, Tianbao Song, Min Zhang, Li Quan, Shengbin Li, Zhong Sheng Sun

**Affiliations:** 1 Department of Forensic Science, School of Medicine, Xi'an Jiaotong University, Xi'an, Shan'xi, China; 2 Key Lab of Mental Health, Institute of Psychology Chinese Academy of Sciences, Beijing, China; 3 Department of Molecular Immunology, Capital Institute of Pediatrics, Beijing, China; 4 Beijing Institutes of Life Science, Chinese Academy of Sciences, Beijing, China; 5 Institute of Genomic Medicine, Wenzhou Medical College, Wenzhou, China; Radboud University, The Netherlands

## Abstract

Analysis of global methylation in cells has revealed correlations between overall DNA methylation status and some biological states. Recent studies suggest that epigenetic regulation through DNA methylation could be responsible for neuroadaptations induced by addictive drugs. However, there is no investigation to determine global DNA methylation status following repeated exposure to addictive drugs. Using mice conditioned place preference (CPP) procedure, we measured global DNA methylation level in the nucleus accumbens (NAc) and the prefrontal cortex (PFC) associated with drug rewarding effects. We found that cocaine-, but not morphine- or food-CPP training decreased global DNA methylation in the PFC. Chronic treatment with methionine, a methyl donor, for 25 consecutive days prior to and during CPP training inhibited the establishment of cocaine, but not morphine or food CPP. We also found that both mRNA and protein level of DNMT (DNA methytransferase) 3b in the PFC were downregulated following the establishment of cocaine CPP, and the downregulation could be reversed by repeated administration of methionine. Our study indicates a crucial role of global PFC DNA hypomethylation in the rewarding effects of cocaine. Reversal of global DNA hypomethylation could significantly attenuate the rewarding effects induced by cocaine. Our results suggest that methionine may have become a potential therapeutic target to treat cocaine addiction.

## Introduction

Drug addiction is defined as a compulsive pattern of drug-seeking and drug-taking behavior that craving continues despite adverse physical or psychosocial consequences [1]. These behavioral abnormalities develop gradually and progressively following repeated exposure to drugs of abuse, and can persist for months or years after discontinuation of drug use [2]. It is suggested that drug-induced neural plasticity drives these long-term behavioral abnormalities and changes of gene expression in brain reward regions playing important roles in this process [1], [2], [3]. However, the molecular mechanisms underlying drug-induced stable alterations in gene expression are still unknown. Accumulating evidence demonstrated that epigenetic modification, including histone acetylation and DNA methylation, plays an important role in gene regulation induced by additive drugs [4]. Acute exposure to cocaine increases the level of histone H4 acetylation on the proximal gene promoters of immediate early genes *c-fos* and *Fosb*. In addition, repeated cocaine exposures increase histone H3 acetylation on the gene promoters of both *Cdk5* (Cyclin-dependent kinase 5) and *Bdnf* (brain derived neurotrophic factor) in the nucleus accumbens (NAc) [5]. Furthermore, systemic administration or intra-NAc injection of HDAC inhibitor significantly enhances cocaine reward [5], [6]. In the prefrontal cortex (PFC), *Npy* (neuropeptide Y) expression was upregulated and its promoter was hyperacetylated after withdrawal from cocaine self-administration; whereas *Egr-1* (early growth -1) was found to be downregulated and hypoacetylated under the same condition [7]. Significant reductions were also evident in global levels of both the H_3_k_4_ me^3^ and H_3_K_27_me^3^ in the PFC after exposure to cocaine in adolescence rats. It may indicate roles of chromatin remodeling in the observed changes in genes encoding cell adhesion molecules and transcription factors [8].

DNA methylation is another key epigenetic mechanism in regulation of gene expression. It occurs by transfer of a methyl group from S-adenosyl methionine (SAM), a universal methyl group donor in biochemical reactions in cells, to cytosine residues at the dinucletide sequence CpG, and is catalyzed by DNA methytransferases (DNMTs) [9]. The best documented function of DNA methylation in specific regulatory regions is in silencing gene expression by either providing targets for enzymes that modify the chromatin to be in a closed and inactive configuration [10] or by blocking the interaction of transcription factors with regulatory regions of the gene [11], [12]. Recent results have indicated association between the status of DNA methylation in promoter region of several genes and drug addiction. For example, DNA hypermethylation in the *Oprm1* promoter region was demonstrated in lymphocytes of former methadone-maintained heroin addicts [13]. It is found that acute and repeated cocaine treatments result in DNA hypermethylation and transcriptional downregulation of the protein phosphatase-1 catalytic subunit (*Pp1c*) gene, and hypomethylation and transcriptional activation of *FosB* gene in the NAc [14]. More recently, using conditioned place preference (CPP) paradigm in marmoset monkeys, the reduction of DNA methylation at a specific CpG site neurokinin3-receptor (NK3-R)-receptor (TACR3) coding genes was also reported [15]. In addition, global DNA methylation in cells or tissues have revealed correlations of overall methylation status with cancer and aging, suggesting that global DNA methylation may be a hallmark in certain biological processes and diseases [16]. However, there is no investigation to detect the global status of DNA methylation after exposure to addictive drugs.

CPP is a Pavlovian procedure in which one context is paired with drug injections during the training phase, while another context is paired with vehicle injections [17], [18]. During subsequent drug-free CPP test, animals choose between the drug- and vehicle-paired contexts. Increased preference for the drug context serves as measures of drug reward [19] and incentive motivational effects of drug cues [20]. In the current study, using CPP procedure, we examined the role of the global DNA methylation level in the NAc and the PFC in regulating drug rewarding effect. Then, we investigated potential roles of DNMTs and MBD2 in regulating DNA methylation underlying drug rewarding effects.

## Results

### Cocaine but not morphine or food CPP induced global DNA hypomethylation in the PFC

**Figure 1:**
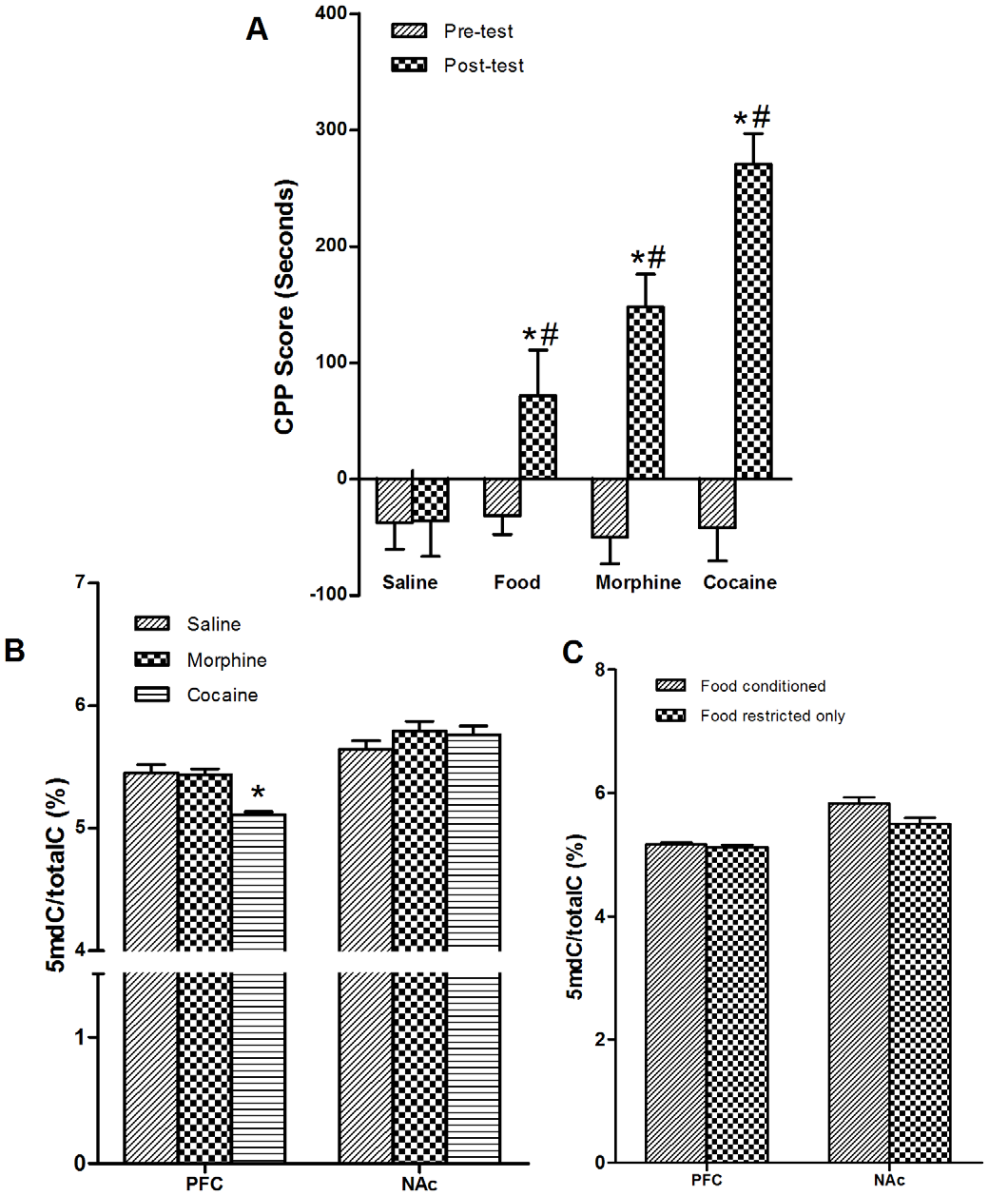
Changes in global DNA methylation by cocaine-, morphine-, and food-CPP. (a) Establishments of food-, morphine- and cocaine-CPP. *p<0.05, postconditioning in comparison with preconditioning within the same group; # *p*<0.05 in comparison with saline group during postconditioning (*n* = 16 for saline; n = 20 for food; n = 18 for morphine, and *n* = 18 for cocaine); (b) Changes of global DNA methylation level in the PFC, and in the NAc following cocaine- and morphine CPP. *p<0.05 in comparison with saline group (*n* = 16 for saline; n = 9 for morphine and cocaine); (c) Food-CPP training did not change the global DNA methylation **(n = 10)**. Data are depicted as the percentage of methylated cytosine in total cytosine. Data are expressed as mean±SEM.

To determine the role of DNA methylation in cocaine, morphine and food CPP, we examined the global DNA methylation in the PFC and the NAc 2 h after CPP testing. Figure 1a showed that there were significant differences for the time spend in the food-, morphine- or cocaine-paired chamber between saline and morphine groups (F_(1,32)_ = 8.69, *p* = 0.006), as well between saline and cocaine groups (F_(1,32)_ = 21.68, *p*<0.001). *Post hoc* analysis revealed that time spent in the morphine- and cocaine-paired chamber were significantly increased after CPP training [*p*<0.001, preconditioning vs. postconditioning for morphine and cocaine groups]. After food CPP training, time spent in the food-paired chamber was significantly increased (t = 2.42, *p* = 0.021). As shown in Fig. 1 b and c, there were significant differences for the global DNA methylation among saline, morphine and cocaine groups (F_(2, 6)_ = 13.86, *p* = 0.006) in the PFC but not in the NAc. *Post hoc* analysis revealed that the global DNA in the PFC was significantly decreased in the cocaine-CPP group (*p* = 0.004, cocaine group vs. saline group). However, there were no significant changes in both the PFC and the NAc after CPP training in food and morphine groups. These results indicated that global DNA hypomethylation was specifically responding to cocaine in certain brain region.

### Systemic administration of methionine prevented the CPP establishment of cocaine but not morphine or food

**Figure 2:**
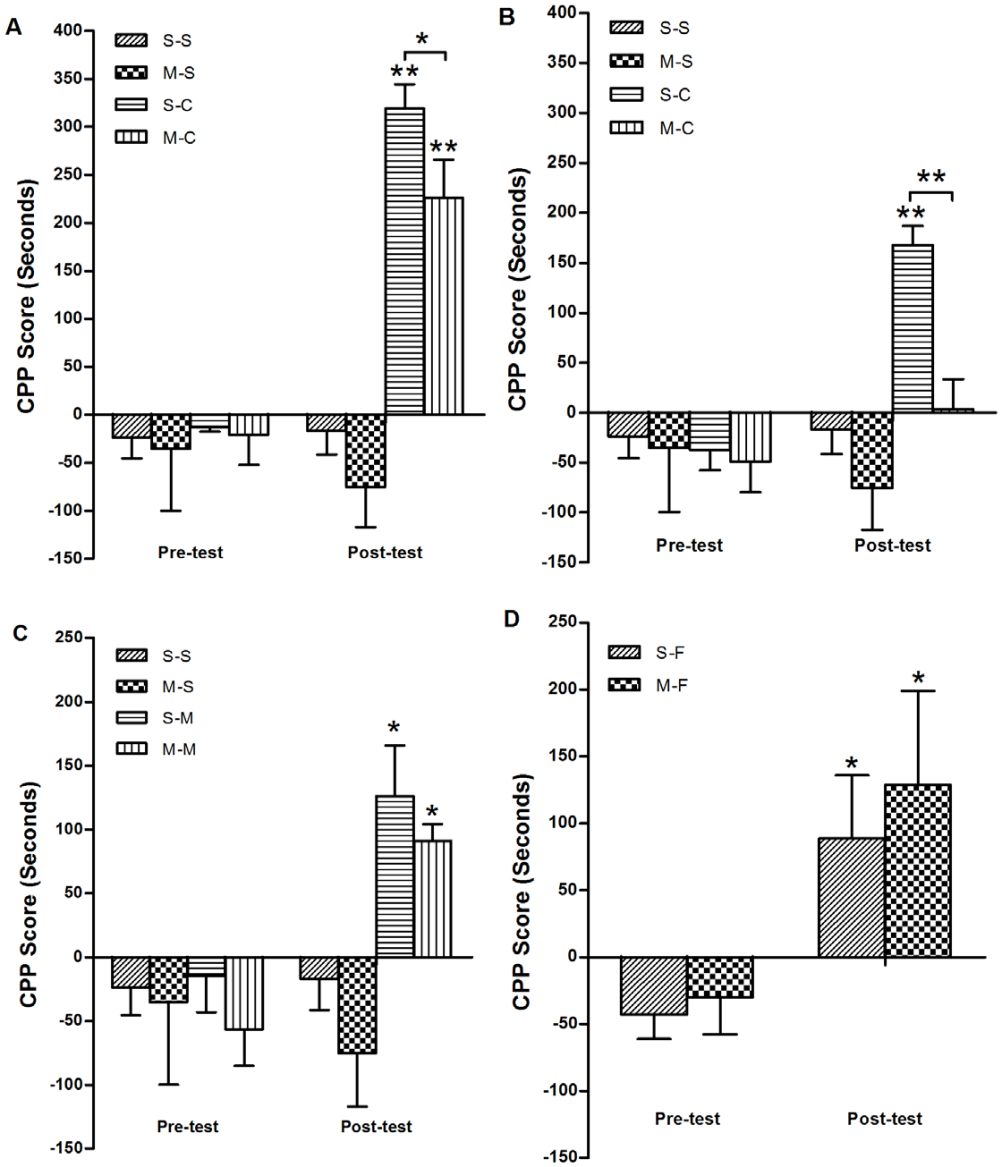
Effects of chronic methionine treatment on the establishment of cocaine-, morphine- and food-CPP. Mice were injected subcutaneously with 1 g/kg (6.6 mmol/kg) L-methionine twice a day for 25 consecutive days before (15 d) and during CPP procedures (10 d) respectively. (a) Methionine significantly inhibited the establishment of cocaine-CPP at 20 mg/kg cocaine; (b) Methionine abolished the establishment of cocaine-CPP at 5 mg/kg cocaine; (c) Methionine did not affect the establishment of morphine-CPP; (d) methionine did not affect the establishment of food-CPP. *p<0.05 and ** *p*<0.001, pretreatment in comparison with posttreatment; **p<0.001, postconditioning in comparison with preconditioning within the same groups. Data are expressed as mean ± SEM. [S-S: n = 8; M-S: n = 7; S-C: n = 10(20 mg/kg); n = 7(5 mg/kg); M-C: n = 10(20 mg/kg) n = 6(5 mg/kg); S-M: n = 10; M-M: n = 8; S-F: n = 10; M-F: n = 8].

To determine the effects of methionine treatment on rewarding response to food, morphine and cocaine, mice were administrated methionine systemically for 15 and 10 consecutive days prior to and during CPP training. As shown in Figure 2a and 2b, one-way ANOVA analysis showed that there were significant differences in cocaine induced CPP among groups of saline and cocaine CPP with or without methionine treatment (F_(3,31)_ = 14.08, *p*<0.001 for higher dose cocaine; F_(3,24)_ = 3.89, *p* = 0.02 for lower dose cocaine). *Post hoc* analysis revealed methionine administration significantly attenuated cocaine-CPP establishment at higher dose (20 mg/kg) (S-C vs. M-C *p* = 0.04) and completely abolished cocaine-CPP expression at lower dose (5 mg/kg) (S-C vs. M-C *p*<0.001). However, methionine treatments had no effects on CPP establishment of morphine or food (Figure 2c and 2d).

### Cocaine induced hypomethylation in the PFC could be reversed through methionine administration

**Figure 3:**
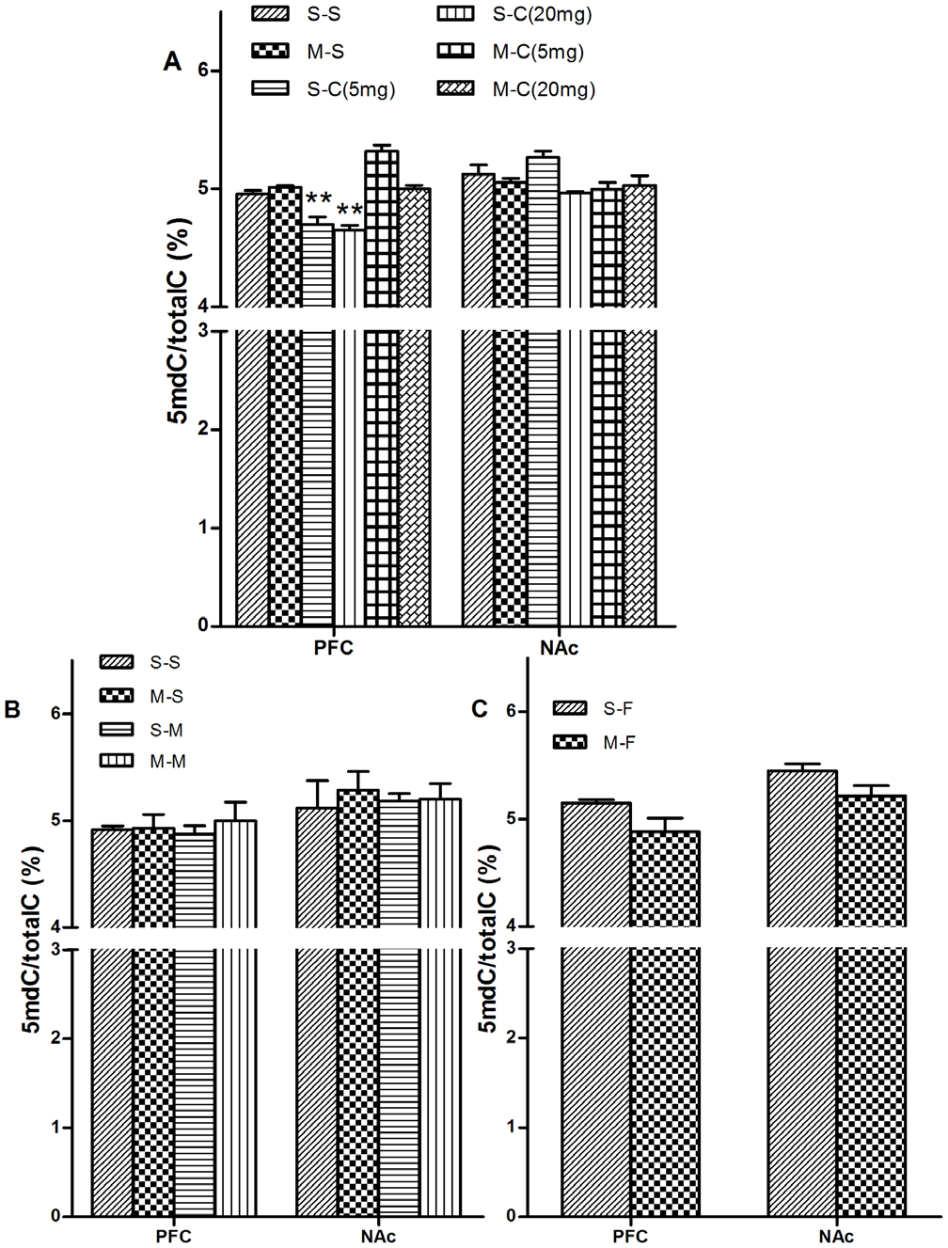
Effects of methionine on global DNA hypomethylation. (a) Methionine reversed global DNA hypomethylation induced by cocaine-CPP; (b) Methionine did not affect global DNA methylation in morphine-CPP groups; (c) Methionine did not affect global DNA methylation in food-CPP groups. *p<0.05 in comparison with S-S group. Data are expressed as mean ± SEM. [S-S: n = 8; M-S: n = 7; S-C: n = 10(20 mg/kg), n = 7(5 mg/kg); M-C: n = 10 (20 mg/kg), n = 6(5 mg/kg); S-M: n = 10; M-M: n = 8; S-F: n = 10; M-F: n = 8].

As shown in Figure 3a, one-way ANOVA analysis revealed that there were significant differences in global DNA methylation level in the PFC but not in the NAc among S-S, M-S, S-C and M-C groups (F_(5,12)_ = 8.91, *p*<0.001). Consistent with the behavioral test, *Post hoc* analysis showed that the global DNA hypomethylation induced by cocaine CPP in the PFC were reversed [S-C (5 mg/kg) vs. M-C (5 mg/kg) *p*<0.001; S-C (20 mg/kg) vs. M-C (20 mg/kg) *p* = 0.001 respectively]. These results indicate the crucial roles of global DNA hypomethylation in the PFC and cocaine-CPP establishment. As shown in Figure 3b and 3c, methinonine did not increase global DNA methylation in the PFC associated with food and morphine CPP.

### Effects of methionine on the mRNA and protein expression of DNMTs and MBD2

**Figure 4:**
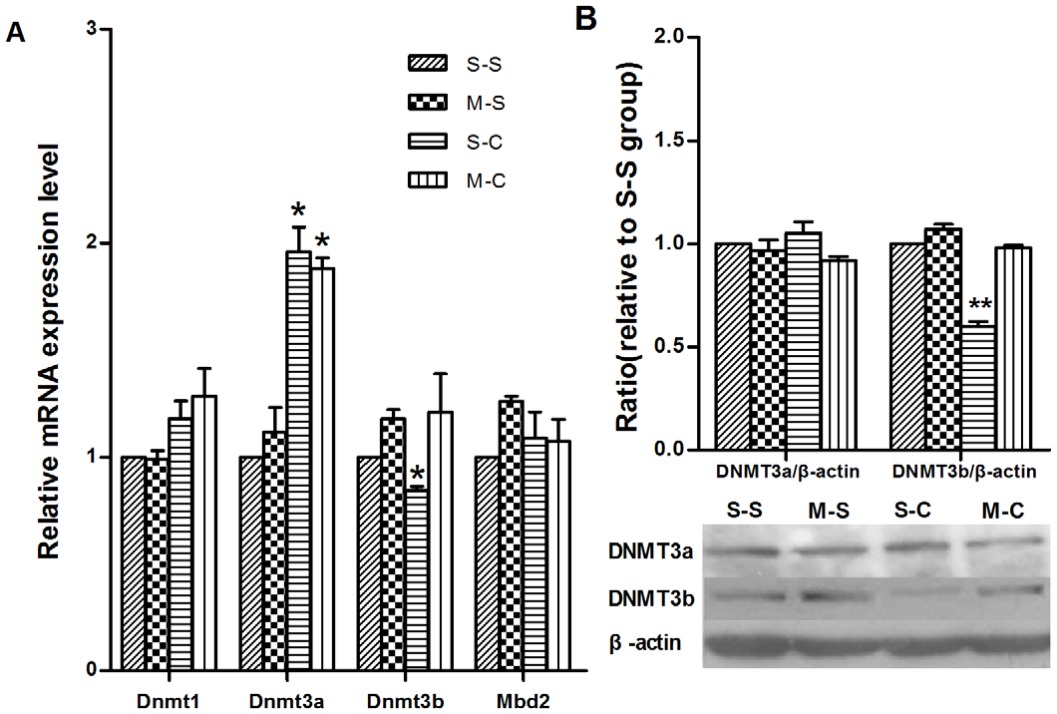
Effects of methionine on the mRNA and protein expression of DNMTs. (a) The effects of methionine on the mRNA expressional alteration of *Dnmt*s and *Mbd2* induced by cocaine-CPP. Data depicted as the relative gene expression level. (b) The effects of methionine on the alteration of DNMT3a and 3b induced by cocaine-CPP. Data depicted as the relative protein expression level. *p<0.05 in comparison with S-S group. Data are expressed as mean±SEM. [S-S: n = 8(Fig. a), n = 10(Fig. b); M-S: n = 7(Fig. a), n = 9(Fig. b); S-C: n = 10(Fig. a), n = 9(Fig. b); M-C: n = 10(Fig. a), n = 8(Fig. b)].

To date, increasing data proposed that DNA methylation is a reversible reaction. The steady state of DNA methylation status of a gene reflects a balance of methylation and demethylation [21]. Thus, we speculated that cocaine exposures could disturb this balance through the stimulation of demethylation or inhibition of methylation. Both processes may further result in global DNA hypomethylation. It is generally accepted that DNA methylation occurs through catalysis of DNMTs such as DNMT1, DNMT3a and DNMT3b [21]. Recently, methylated DNA-binding protein 2 (MBD2) was shown to directly remove the methyl group from methylated cytosine in methylated CpGs [22]. Moreover, it was proposed that the DNA methyltransferase DNMT3a acts as a demethylase, possibly through a mechanism that involves deamination [23]. To understand the mechanisms of methionine treatment underlying the behavioral response to cocaine, we compared the mRNA expression of *DNMTs* and *Mbd2* with methionine treatment to the one without methionine treatment. As shown in Figure 4a, *Dnmt1* and *Mbd2* were not significantly changed in response to methionine treatment. There were significant differences for mRNA expression of *Dnmt3a* and *3b* among S-S, M-S, S-C and M-C groups (F_(3, 8)_ = 30.54, *p*<0.001 for *Dnmt3a* and F_(3, 8)_ = 8.23, *p* = 0.04 for *Dnmt 3b*). *Post hoc analysis* revealed that mRNA expression of *Dnmt3b* (S-S vs. S-C *p*<0.05) was downregulated following cocaine CPP training at 20 mg/kg cocaine. The downregulation of *Dnmt3b* (S-C vs. M-C *p*<0.05) was also reversed by methionine administration. Although *Dmnt3a* mRNA [S-C vs. M-C *p*<0.001] was upregulated following cocaine CPP training, the upregulation of *Dnmt3a* [S-C vs. M-C *p* = 0.548] was not reversed by subsequent methionine administration.

As the level of protein rather than mRNA expression represents the key roles in the cell [24], protein levels of DNMT3a and DNMT 3b were also measured in the PFC by western blotting. As shown in Fig. 4b, the protein level of DNMT3b (S-S vs. S-C *p*<0.001) was downregulated following cocaine CPP training, and the downregulation of DNMT3b (S-C vs. M-C *p* = 0.004) was reversed under methionine administration. However, the DNMT3a protein failed to show any significant differences among all groups despite of the changes in its mRNA level described above. These results further demonstrated that DNMT3b but not DNMT3a played an important role in cocaine CPP and methionine effects on cocaine CPP.

It is reported that MeCP2 (methyl CpG binding protein 2) regulates the cocaine effects [25]. However, we didn't find the alteration of MeCP2 in mRNA level in the PFC (Figure S1). Furthermore, we detected mRNA expression of some potential target genes which may be related to cocaine CPP. As shown in Figure S2, there were no significant differences of mRNA expression of *Bdnf* (brain-derived neurotrophic factor), *Reelin* and *Gad-1*(glutamic acid decarboxylase 1) in the PFC after cocaine-CPP training.

## Discussion

The main findings of the present study were as below: CPP induced by cocaine, but not by morphine or food, decreased global DNA methylation in the PFC, but not in the NAc. Secondly, chronic treatment of methionine, the precursor of SAM and the methyl donor of DNA methylation reaction, significantly attenuated the establishment of CPP at a higher dose of cocaine and abolished the establishment of CPP at lower dose cocaine. In contrast, the same treatment did not significantly affect the establishment of morphine- or food-CPP. Meanwhile, the hypomethylation of global DNA in the PFC induced by cocaine CPP was also reversed by repeated administration of methionine. Thus, global DNA hypomethylation in the PFC was associated with the rewarding effect of cocaine. Finally, both mRNA and protein level of DNMT3b were downregulated following cocaine-CPP and this change was reversed by chronic methionine treatment.

### DNA hypomethylation in the PFC contributes to cocaine rewarding effect

The PFC is considered to be a critical area for the development and maintenance of addiction-related behaviors such as CPP [8]. For example, specific lesion of the prelimbic mPFC was sufficient to block cocaine induced CPP [26], [27]. Furthermore, noradrenergic and 5-HT depletion of the PFC could attenuate the preference for cocaine-associated environment [28], [29]. In the present study, we found that cocaine CPP resulted in global DNA hypomethylation in the PFC. It indicates the involvement of DNA methylation in the molecular mechanisms underlying cocaine-reward-related processes. In line with our results, it has been reported that DNA methylation at a specific CpG site within TACR3 coding genes was decreased after CPP training in marmoset monkeys [15]. In normal cells, most methylcytosine is not located in DNA sequence of gene body (less 5% DNA), but rather in repetitive sequences that constitute about half of the genome [30]. The decrease of DNA methylation in the genome may result from hypomethylation in both genes and the CpG-rich noncoding components of genome [16], [30]. Our results found that cocaine specifically induced global DNA hypomethylation, which may reflect the changes associated with the methylation in CpG dispersed throughout repetitive sequences as well as the bodies of genes.

What are the potential mechanisms to regulate cocaine-triggered hypomethylation in the PFC? Accumulating evidence showed that patterns of DNA methylation might be reversible even in adult neurons [31]. The steady state of DNA methylation status reflects a balance of methylation and demethylation [22]. DNA methylation is catalyzed by DNMTs exclusively [21], whereas the demethylation was may catalyzed by MBD2 [22] and DNMT3a [23]. It is possible that DNA hypomethylation following cocaine CPP training is caused either by excessively passive demethylation or excessive inhibition of methylation. This process occurs partly via impairment of equilibrium of DNMTs and demethylase activity. Previous study showed that DNMT3b deficiency is responsible for global hypomethylation in the immunodeficiency, centromeric instability, and facial anomalies syndrome [16]. In the current study, we found that *Dnmt3b* was downregulated in both mRNA and protein level, indicating its potential role in PFC global DNA hypomethylation induced by cocaine CPP.

Our results proved that the inhibitory effects of methionine treatment on the establishment of cocaine CPP may result from the reversal of global DNA hypomethylation in the PFC. The effect of methionine was specific to the establishment of cocaine CPP since the same treatment didn't affect the morphine or food CPP. Furthermore, morphine or food CPP training did not produce global DNA hypomethylation. The observation that methionine had no inhibitory effect on the food preference test suggested that methionine did not produce anhedonia, which did not result in impairment of general learning and memory. After methionine treatment, the downregulation of DNMT3b was reversed. These results suggest that methionine may inhibit cocaine CPP via DNA re-methylation mediated by increased activity of DNMT3b.

### Differences among cocaine, morphine and natural reward

As a matter of fact, there are many neurobiological differences between natural and drug-related rewards. For example, repeated electrical stimulation of reward-related brain regions affects cocaine but not natural reinforcement [32]. A recent study has also demonstrated that histone acetylation is involved in regulating gene transcription in cocaine but not sucrose self-administration [33]. Furthermore, HDAC activity in the PFC is significantly inhibited by cocaine but not by sucrose self-administration. HDAC inhibitors have also been found to decrease the motivational effect of cocaine but not sucrose [33]. Our results extended previous studies by demonstrating that alterations of global DNA methylation significantly differed in the cocaine and natural reward.

Different pharmacological mechanisms are underlying a wide range of behavioral effects of morphine and cocaine. Cocaine directly targets dopamine transporter and increase extracellular dopamine concentrations. In contrast, morphine indirectly activates VTA dopamine neurons by binding to opioid receptors on tonically active neighboring gamma-aminobutyric acid (GABA) interneurons and thus alleviating this local inhibition [34], [35]. In addition, molecular pathways including GR, neurokinen-1 receptor [34], [36] involved in morphine and cocaine effects were also distinct. Therefore, it is possible that cocaine and morphine exert differential effects on global DNA methylation, although the precise molecular mechanisms underlying these differences are still unknown. However, the lack of changes of global DNA methylation observed in our results does not rule out an involvement of DNA methylation in morphine- or food-related reward since both DNA methylation and demethylation events simultaneously participate in the same process such as fear conditioning and acute cocaine treatment [14], [37]. Therefore, the alteration of some specific sites of DNA methylation may not accompany with changes of the global DNA methylation. Further studies need to be done to investigate DNA methylation status at the specific site of DNA in morphine- or food-related reward.

In conclusion, our results demonstrated that global DNA hypomethylation in the PFC plays a crucial role in cocaine-CPP, likely mediated by inhibition of DNA methylase (DNMT3b). Chronic methionine treatment inhibited the establishment of cocaine-CPP through DNA re-methylation mediated by stimulating DNMT3b. We suggest that reversal of DNA demethylation could have potential therapeutic target treat cocaine addiction.

## Materials and Methods

### Animals

All the mice were purchased from the Laboratory animal center of Academy of Military Medical Science. Adult male C57/BL6 mice (20–30 g) were housed under a 12 h light/dark cycle with supplementary of food and water in *ad libitum* except for mice trained food CPP. To make the experimental condition comparable with food restricted mice, all the mice were single housing. All protocols for animal procedures were approved by Animal Usage Committee of the Institute of Psychology, CAS in compliance with the institutional, local, and international guidelines.

### Drugs

The standard 5-methyl-2-deoxycytidine (5mdC) was purchased from TCI (Tokyo, Japan). Snake venom phosphodiesterase and the other 9 standards including deoxycytidine (dC), deoxyguanosine (dG), deoxyadenosine (dA), thymidine (T), 5-methyl-2-cydine (5mC), cytidine (C), guanosine (G), adenosine (A) and uridine (U) was obtained from Sgima Aldrich (St. Louis, MO, USA). Benzonase, and HPLC grand methanol were bought from Merck (Darmstadt, Germany). Alkaline phosphatase was purchased from Sangon Biotech Co. Ltd Formic acid was get from Dikma Technologies Inc (Beijing, China).

For methionine (Sigma, USA) experiments, mice were injected subcutaneously with 1 g/kg (6.6 mmol/kg) L-methionine twice a day for 25 consecutive days prior to (15d) and during CPP (10d) including preconditioning, conditioning and testing (posttreatment). This was an effective dose and duration shown to increase methylation level at DNA certain regions in the brain [38]. During CPP procedure, mice were injected with methionine 1 h before behavioral experiments.

### Conditioned place preference (CPP)

The apparatus for CPP conditioning and testing were three-chamber polyvinyl chloride (PVC) boxes. Two large side chambers (15 cm long·15 cm wide·15 high Cm) were separated by a smaller one (15 cm long·5 cm wide·15 cm high with smooth PVC floor) with differences in floor texture (bar or grid, respectively). Three distinct chambers were separated by manual guillotine doors and were equipped with infrared photobeams connected to a computer that recorded the mice location in the chambers.

An unbiased conditioning protocol was used as described previously [39]. To determine baseline place preference, the mice were initially placed in the middle chamber with the doors removed for 15 min (preconditioning), and then arranged into control and experimental groups with equivalent preconditioning test scores. During the subsequent conditioning days, mice were paired for 8 d (4 sessions) with the saline group receiving saline in both sides of the chambers. Drug groups were injected drugs [morphine (10 mg/kg.s.c.) or cocaine (5, 20 mg.kg.i.p.)] and saline alternately on one of the sides and saline on the opposite side [33]. Mice were restricted to food one week before training for food-CPP, and induce a loss of 15% of the original body weight [40]. During the conditioning phase, mice received food on one of the sides and received nothing on the opposite side. After each manipulation, the mice were confined to the corresponding conditioning chambers for 30 min for cocaine CPP or 50 min for food and morphine CPP before returning to their home cages. After conditioning, the mice were tested the establishment of CPP (postconditioning/posttreatment) under the same conditions as those described for the preconditioning test. A place preference score (CPP score) was assigned by subtracting the time spent in the drug (food)-paired chamber from the time spent in the saline (no food)-paired chamber.

### Experimental groups and tissue sample collection

Total of three distinct batches of mice were used in the present study. In the first batch, the mice were conditioned saline, morphine, cocaine and food CPP. Two hours after CPP testing (postconditioning), the mice were decapitated after CO_2_ anesthesia. Then the PFC was directly dissected, and the NAc was punched from 1-mm-thick coronal slices using 1.1–1.2-mm internal diameter glass capillaries. The changes of global DNA methylation were examined in both NAc and PFC. In the second batch, the mice were pretreated with methionine or saline, and then conditioned saline morphine, cocaine and food CPP. The tissues of PFC and NAc were collected 2 hours after CPP testing (posttreatment), and then mRNA expression (*Dnmts*, *Mbd2*, *Bdnf*, *MeCP2*, *Reelin* and *Gad-1*) and level of global DAN methylation were examined. In the third batch, after pretreating with methionine or saline, the mice were conditioned saline and cocaine CPP, then the tissues were collected and the changes of protein expression of DNMTs were examined.

### Global DNA methylation analysis

The global measurements of DNA methylation were performed as described previously [41] with some modifications. DNA was hydrolyzed to deoxyribonucleosides before LC separation that based on the procedure reported by Quinlivan [42]. Briefly, digestion solution was composed with 250 U Benzonase, 300 mU phosphodiesterase I, and 200 U alkaline phosphatase in 5 ml Tris–HCl buffer (pH 7.9, 20 mM) containing 100 mM NaCl and 20 mM MgCl_2_. 100 ng (10 ng/uL) DNA samples were digested by adding 10 µL of the digestion solution for incubating at 37°C for 6 h. The 10 uL hydrolysis product was diluted with 250 uL ultra-pure water before sample injection. Then, the products were analyzed for global DNA methylation on An Agilent 1200 series rapid resolution liquid chromatography-QQQ mass spectroscopy system (Palo Alto, CA, USA). An Agilent AQ-C18 50 mm×2.1 mm i.d. (3.5 µm particle size) column was used for separation. The mobile phase A and B containing 0.1% formic acid in water and 0.1% formic acid in methanol were sequentially injected with a 10 uL volume and a 0.2 mL/min flow rate. MRM mode was chosen for monitoring the transition pair of the three target molecular m/z 242.2→126.1, 228.1→112.1, 268.2→152.1 for 5mdC, dC, and dG respectively in method validation and DNA sample quantification. DNA methylation level was calculated from a calibration curve ranged from 1% to 10% for methylation level ranged from 2% to 6% in mammalian genomes. The calibration curve was obtained by plotting the ratio of the peak area of 5mdC to the natural internal standard dG against the concentration ratio of 5mdC to dG.

### RNA isolation and real-time PCR

Total RNA and DNA were extracted using DNA/RNA isolation kit (Tiangen, China) following the manufacturer's instructions, and 1.0 ug total RNA was reverse-transcribed into single-strand cDNA using Superscript III (Invitrogen, USA). Real-time PCR was performed on an Opticon 2 real-time PCR machine (BIO-RAD, U.S.A.) using SYBR Green mix (Tiangen, China) with the following cycling program: 95°C for 10 min, 40 cycles of 95°C for 25 s, 60°C for 25 s, and 72°C for 25 s. The aliquot of cDNA was amplified with a pair of primers as shown in Table 1 and Table S1. G*adph* was used as an internal control for normalization. Each sample was run in triplicates.

**Table 1:** Sequence of primers used for RT-PCR.

Gene	Sequence of primer (5′-3′)	
	Forward	Reverse
Dnmt1	GGAAGGCTACCTGGCTAAAGTCAAG	ACTGAAAGGGTGTCACTGTCCGAC
Dnmt3a	TTCTTGAGTCTAACCCCGTGATG	CTTGCAGCTCCAGCTTATCATTC
Dnmt3b	AGTGACCAGTCCTCAGACACGAAG	ATCAGAGCCATTCCCATCATCTAC
Mbd2	CTGGCAAGATACCTGGGAAA	TTCCGGAGTCTCTGCTTGTT
Gadph	TGCACCACCAACTGCTTA	GGATGCAGGGATGATGTTC

### Western blotting

Grinded in liquid nitrogen, PFC tissues were then incubated for 30 min in ice-cold lysis buffer(10×lysis buffer: Tris-HCl 20 mmol/L(pH 7.5), NaCl 150 mM, EDTA 1 mM, EGTA 1 mM, TritonX-100 1%, Sodium pyrophosphate 2.5 mM, β-Glycerrophosphate 1 mM, NaVO4 1 mM, Leupeptin 1 ug/ml. diluted to 1×lysis buffer before adding 1 mM PMSF) containing protease and phosphatase inhibitors, After concentrated at 12,000 rpm for 5 min,the supernatant was then aliquotted and stored at −80°C. Protein concentration was quantified using the BCA Protein Assay. 50 µg of whole lysates were run on 10% SDS denaturing polyacrylamide gels with 80 V for 30 min followed by 120 V for1.5 h and transferred onto polyvinylidene difluoride (PVDF) membranes at 110 mA for 1 h at room temperature. Following transfer, blots were blocked (10% skimmed milk in TBS) at 37°C for 30 min .and incubated overnight at 4°C in blotto buffer(1×TBS) with antibodies against DNMT3a (D23G1) Rabbit mAb (1∶800, CST #3598,USA), DNMT3b antibody (1∶800, Abgent AJ1244a,USA), and β-actin(1∶1000, Abgent AM1021b,USA) as a loading control. After primary antibody binding, blots were washed in TBS for 10 min (4 times). After washing, blots were incubated with the secondary antibodies (1∶1000, KPL, 074-1506, USA) conjugated with horseradish peroxidase for 1 hr at room temperature with agitation. Protein bands were analyzed using Phoretix 1D Software

### Data analysis

All behavioral data were displayed as mean±SEM. The morphine and cocaine CPP scores were analyzed using two-way repeated ANOVAs followed by LSD *post hoc* with the between-subjects factors of treatment (saline or drug) and the within-subjects factor of test condition (preconditioning, postconditioning, or posttreatment). The food CPP score were analyzed using t-test. The expression levels of genes after saline and cocaine CPP were analysis using One-sample t-tests. The one-way ANOVAs were used to analyze the difference of global DNA methylation level, mRNA and protein expression after saline or methionine pretreated.

## Supporting Information

Figure S1 **The effects of methionine treatment on the mRNA expression of Mecp2 (methyl CpG binding protein 2) induced by cocaine-CPP training.** Data depicted as the relative gene expression level (SD±SEM).
(TIF)

Figure S2 **Changes in Bdnf (brain-derived neurotrophic factor), Reelin and Gad-1(glutamic acid decarboxylase 1) expression in the PFC induced by cocaine-CPP group.** Data depicted as the relative gene expression level (SD±SEM).
(TIF)

Table S1 **Sequence of primers used for RT-PCR.**
(DOC)
