# Supplementary figures and images for: Reversal of Cocaine-Conditioned Place Preference through Methyl Supplementation in Mice: Altering Global DNA Methylation in the Prefrontal Cortex
Source: PLoS One. 2012 Mar 16;7(3):e33435. doi: 10.1371/journal.pone.0033435 (PMC3306398; doi:10.1371/journal.pone.0033435)

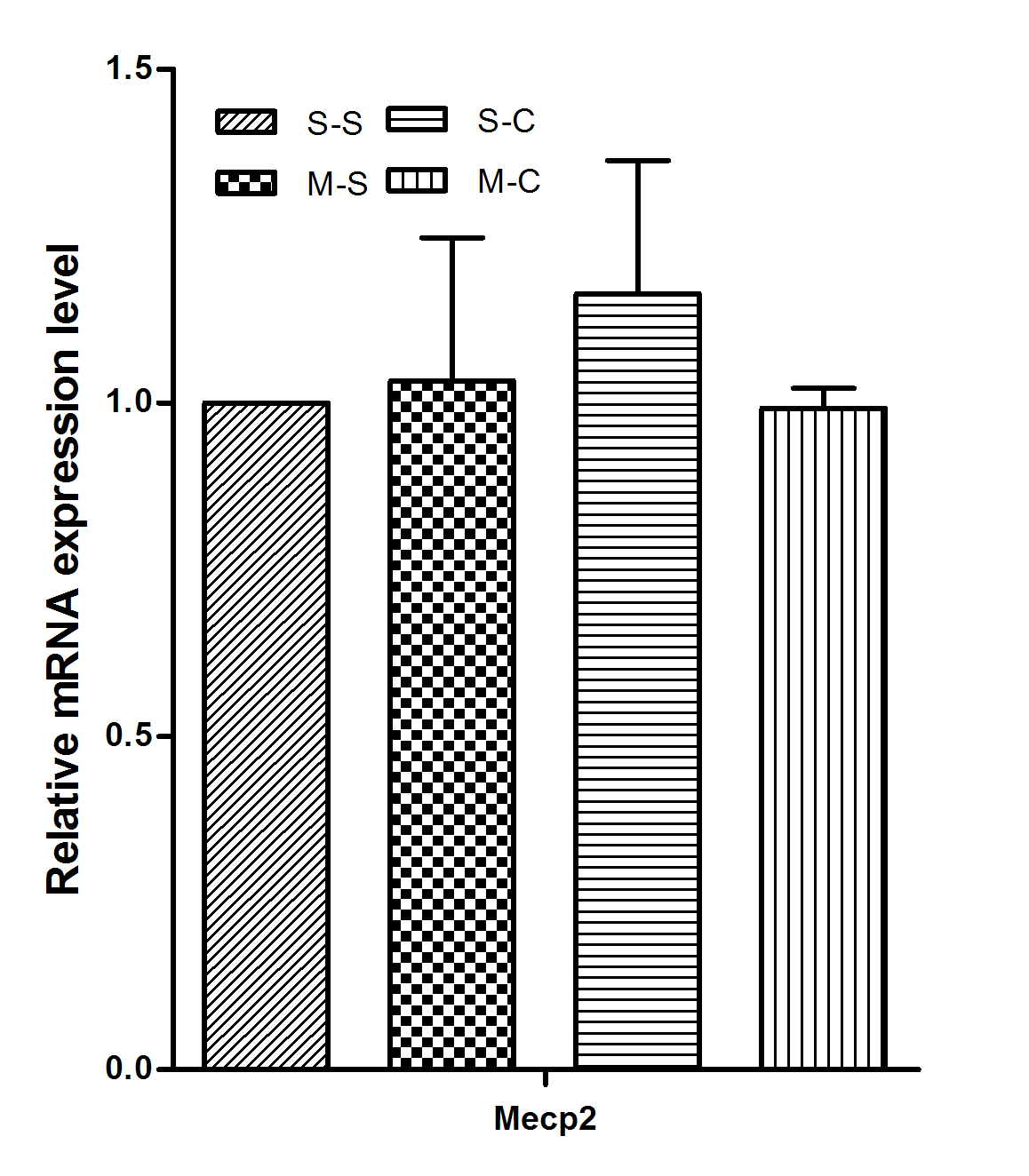

Supplement: Figure S1 — The effects of methionine treatment on the mRNA expression of Mecp2 (methyl CpG binding protein 2) induced by cocaine-CPP training. Data depicted as the relative gene expression level (SD±SEM). (TIF) [file pone.0033435.s001.tif]

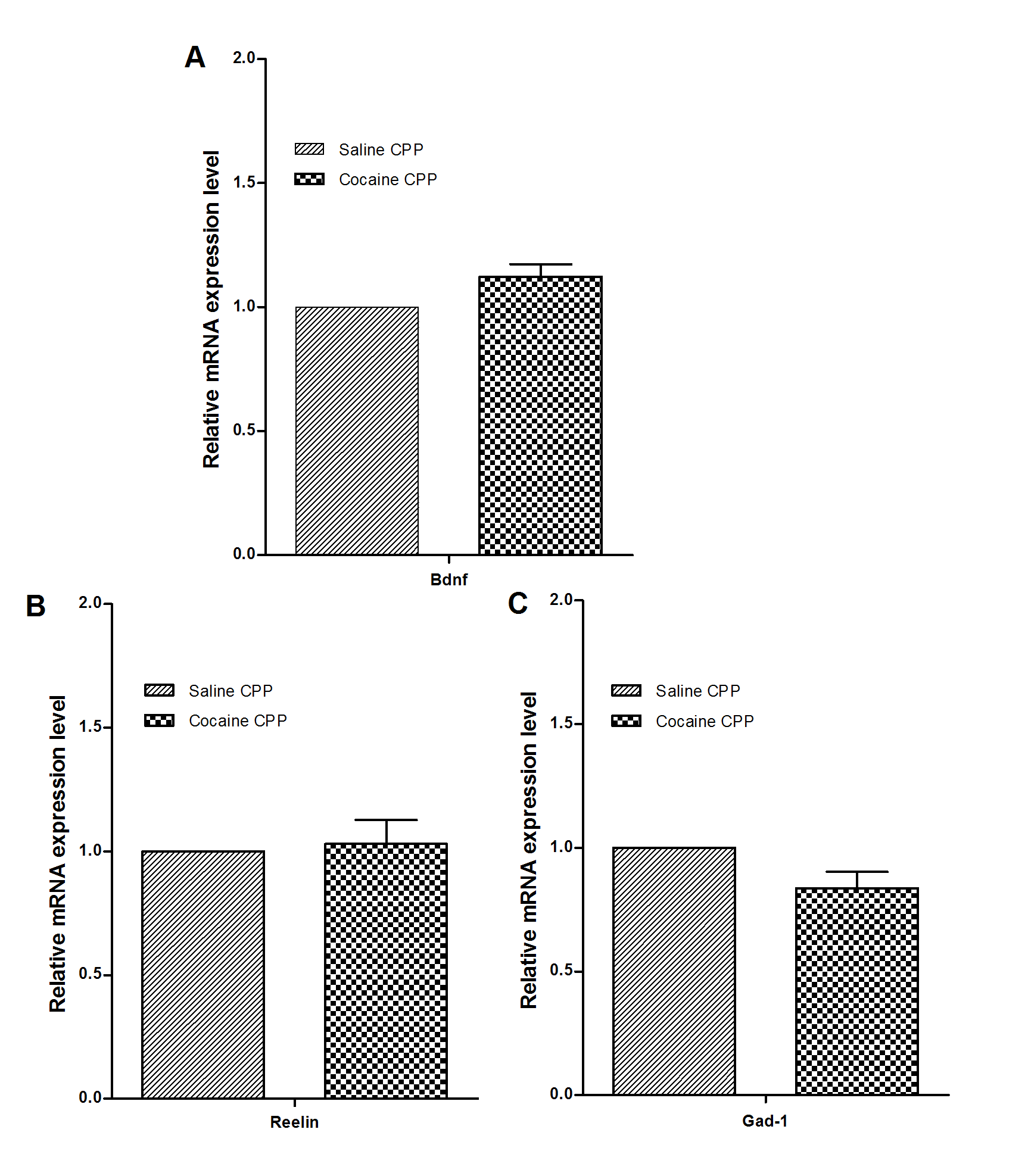

Supplement: Figure S2 — Changes in Bdnf (brain-derived neurotrophic factor), Reelin and Gad-1(glutamic acid decarboxylase 1) expression in the PFC induced by cocaine-CPP group. Data depicted as the relative gene expression level (SD±SEM). (TIF) [file pone.0033435.s002.tif]
