# Supplementary material for: Reversal of Cocaine-Conditioned Place Preference through Methyl Supplementation in Mice: Altering Global DNA Methylation in the Prefrontal Cortex
Source: PLoS One. 2012 Mar 16;7(3):e33435. doi: 10.1371/journal.pone.0033435 (PMC3306398; doi:10.1371/journal.pone.0033435)
Supplement: Table S1 — Sequence of primers used for RT-PCR. (DOC) [file pone.0033435.s003.doc]

| Gene | Sequence of primer (5’-3’) | |
| --- | --- | --- |
|  | Forward | Reverse |
| Mecp2  Bdnf  Reelin  Gad-1  Gadph | CCGGGGACCTATGTATGATG  TGGCTG ACACTTTTGAGCAC  AAGCTTTGGCAGTGCCAGACT  TCACCCTCGATTTTTCAACC  GCACCACCAACTGCTTA | AGGAGGTGTCTCCCACCTTT  AAGTGTACAAGTCCGCGTCC  AAAGGACGTGATTAGCTGCCG  AACAAACACGGGTGCAATTTGGATGCAGGGATGATGTTC |
